# 3D-Printed Model for Surgical Planning in Diverticular Disease: A Case Report

**DOI:** 10.3390/reports8040222

**Published:** 2025-10-31

**Authors:** Alessandro Gemini, Roberto Cirocchi, Luca Properzi, Francesca Duro, Giovanni Domenico Tebala

**Affiliations:** 1 Department of Digestive and Emergency Surgery, “S. Maria” Hospital, 05100 Terni, Italy; roberto.cirocchi@unipg.it (R.C.); luca.properzi@yahoo.it (L.P.); francesca.duro@aospterni.it (F.D.); g.tebala@aospterni.it (G.D.T.); 2 Department of General Surgery, University of Perugia, 06123 Perugia, Italy

**Keywords:** 3D printing, diverticular disease, surgical planning, laparoscopic surgery, case report, colorectal surgery, hemicolectomy, patient-specific model

## Abstract

**Background and Clinical Significance:** Preoperative planning is crucial for improving surgical safety and outcomes, particularly in minimally invasive surgery, where tactile feedback is absent. Three-dimensional (3D) printing offers patient-specific anatomical models that can enhance surgical planning. Its application in diverticular disease remains underexplored. **Case Presentation:** We present the case of a 65-year-old male with recurrent diverticulitis involving the sigmoid and descending colon. After conservative management of an acute episode, preoperative imaging revealed extensive diverticulosis. A patient-specific 3D-printed model was created from CT images to plan the surgical approach. The model helped determine the need for a left hemicolectomy rather than a simple sigmoidectomy, anticipated technical challenges such as lowering the left colic flexure and ligating the inferior mesenteric artery, and improved patient counseling. The surgery was performed laparoscopically without complications, and the patient was discharged on postoperative day six. Histology confirmed diverticulosis with perivisceritis and reactive lymphadenitis. **Conclusions:** This case demonstrates the potential of 3D printing to optimize surgical planning in diverticular disease, enabling tailored resections and improving operative strategy. Broader adoption may be limited by time and cost but offers clear educational and clinical benefits.

## 1. Introduction and Clinical Significance

The importance of intraoperative safety for both patients and surgeons, along with the concept of “tailor-made surgery,” has become a major topic of surgical research in recent years [1]. Patient-centered preoperative planning is essential to gain accurate knowledge of the target anatomy, helping surgeons during critical phases of the procedure and preventing potential complications [2]. Three-dimensional (3D) printing refers to a wide range of technologies used for producing 3D objects [3]. Additive processes are primarily used, in which successive layers of material are deposited under computer control. This way, objects are created in any shape or geometry. Additive manufacturing technology requires the digital representation of geometric data, which is provided in a stereolithography (STL) format or other additive manufacturing file formats [4]. This technology enables the construction of patient-specific 3D-printed anatomical models, which can be used for various purposes, including the development of new surgical tools, the diagnostic process, and surgical planning [5,6,7]. In addition to standard medical imaging, 3D printing can represent a valuable tool for providing a good representation of the surgical scenario, especially in more complex cases [8]. Furthermore, 3D models offer the surgeon an opportunity to review, plan, and study the procedure in detail, even days before the surgery. Beyond surgical planning, 3D-printed models are increasingly used for simulations and skill training [9]. The disadvantages of this technology include its relatively high cost, the limited availability of biomaterials for medical use, and the complexity of transforming the patient’s anatomical data into a computer model for rendering. Currently, there are few studies on the use of 3D technology in colorectal surgery. In the 2019 systematic review by Emile et al. [10], the authors evaluated the use of 3D printing in colorectal surgery. Recent systematic reviews confirm the expanding clinical role of 3D printing in colorectal surgery [11]. No study has yet evaluated the usefulness of 3D printing in planning colon resection interventions for diverticular disease. We applied 3D printing technology in planning laparoscopic colon resection for patients with recurrent diverticulitis. The goals were to (1) define the optimal resection margins; (2) anticipate intraoperative challenges; (3) compensate for the lack of tactile feedback in laparoscopy; and (4) potentially reduce postoperative complications.

## 2. Case Presentation

### 2.1. Application of 3D Printing to Surgical Planning

With the advent of minimally invasive surgery, left colon resections for tumors are typically performed after marking the lesion with ink during a preoperative colonoscopy. This allows the surgeon to identify the lesion’s position intraoperatively and proceed with a radical oncological resection, ensuring the complete removal of the neoplasm. In the case of benign conditions, such as diverticulosis—which shares characteristics with chronic inflammatory bowel diseases, where multiple segments of the colon may be affected simultaneously and heterogeneously—preoperative marking is not possible. The growing prevalence of minimally invasive surgery has eliminated the surgeon’s ability to rely on intraoperative palpation to detect areas of fibrosis or colonic stenosis, which could guide the extent of the resection. Therefore, we decided to apply 3D printing technology to the preoperative planning process, allowing us to assess the extent of the colonic resection beforehand. This approach reduces the risk of performing inadequate resections, which could lead to persistent postoperative symptoms. Preoperative evaluation in patients with diverticulosis who require elective surgery typically involves CT scans, colonoscopy, and sometimes barium enema. However, as noted in the guidelines, CT imaging may not fully reflect intraoperative findings, particularly regarding fibrosis or the extent of diverticulosis. With the help of 3D reconstructions and printing, it is possible to identify potential anatomical challenges before surgery. By assessing the location and extent of the disease, we can determine the type of resection required—whether a sigmoidectomy or left hemicolectomy is needed—and predict how far the resection may need to extend, possibly involving the transverse colon or rectum. This preoperative planning also helps to determine whether the removal of the left colic flexure will be necessary, whether the inferior mesenteric artery should be preserved or divided, and whether the resection will extend to the extraperitoneal rectum. Better planning not only assists the surgical team but also helps explain the procedure and potential complications to the patient, offering a clearer understanding of what to expect from the surgery.

### 2.2. Case Report

**Figure 1:**
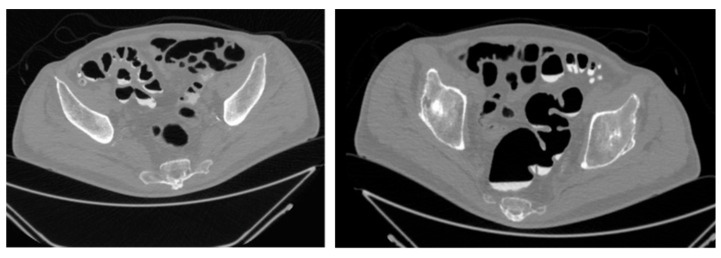
CT—Virtual Colonoscopy: Abdominal CT scan (virtual colonoscopy reconstruction) showing diffuse diverticulosis of the sigmoid colon with associated wall thickening. A perivisceral inflammatory reaction is also evident around the affected area.

**Figure 2:**
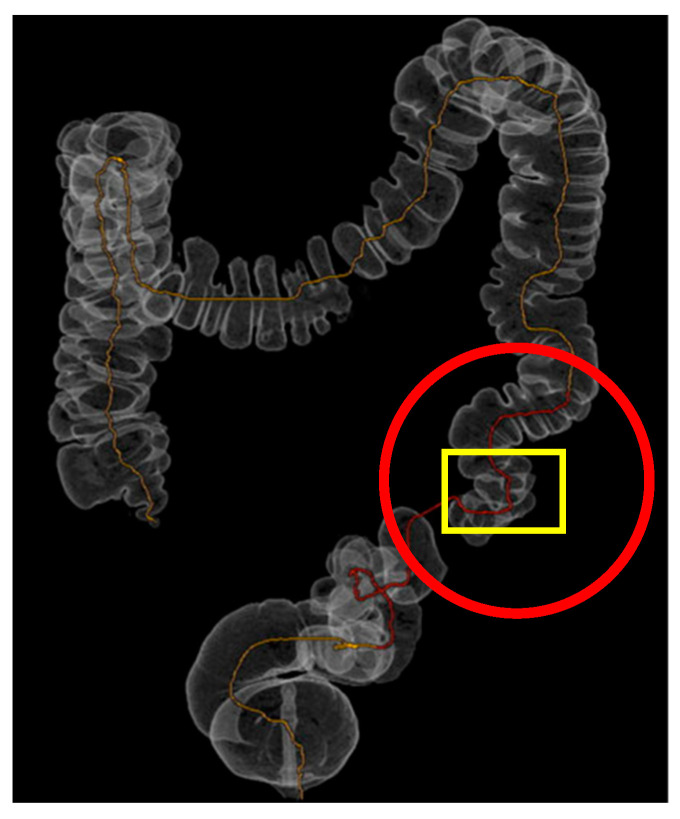
Three-dimensional rendering of CT-colonoscopy: Three-dimensional rendering of CT-colonoscopy (The diverticula are highlighted by red circles, while the stenotic segment is indicated by the yellow box).

A 65-year-old Caucasian male presented to our attention with acute uncomplicated diverticulitis in an emergency setting. The patient had no history of previous surgical interventions and had arterial hypertension. He reported a history of two previous episodes of acute uncomplicated diverticulitis and initially underwent an urgent abdominal CT scan, which showed the following: “In a patient with diffuse diverticulosis, thickening of the sigmoid walls is evident due to perivisceritis, while no signs of free air are detected. Small reactive lymph nodes are present adjacent to this tract, in the absence of peritoneal collections or effusions.” The patient was treated with conservative medical therapy (antibiotics and fluid therapy) and was discharged four days after hospitalization, with a virtual colonoscopy scheduled four weeks later. The virtual colonoscopy confirmed the following: “Massive diverticulosis of the sigmoid colon and descending colon with reduced distensibility of the viscus.” (Figure 1 and Figure 2). Given the patient’s history of recurrent diverticulitis, we decided to propose colonic resection. In planning the surgery, we also opted to use 3D printing to create a preoperative model of the expected intraoperative scenario, allowing for a more precise evaluation of the resection extent.

While the virtual colonoscopy confirmed the presence of massive diverticulosis of the sigmoid and descending colon, the three-dimensional reconstruction more clearly highlighted the presence of a stenotic area within the diseased segment. This additional information was not as evident in the virtual colonoscopy alone and supported the indication for a left hemicolectomy rather than a limited sigmoidectomy.

Based on the three-dimensional reconstruction, the surgical team anticipated the need to mobilize the splenic flexure to achieve a tension-free anastomosis and to ligate the inferior mesenteric artery (IMA), given the extension of the disease. These steps were considered in preoperative planning and were subsequently confirmed intraoperatively.

A comparative summary of the findings obtained from the virtual colonoscopy and from the 3D-printed model is presented in Table 1.

The surgery was performed laparoscopically. The patient underwent a left hemicolectomy with colorectal anastomosis, without a protective stoma. The postoperative course was uneventful, and the patient was discharged on the sixth postoperative day. A histological examination of the surgical specimen revealed the following: “Sections of the large intestine wall with diverticulosis and perivisceritis, as well as reactive lymphadenitis in 10 out of 10 perivisceral lymph nodes. Anastomotic rings free from significant alterations.”

### 2.3. Three-Dimensional Printing Design and Implementation Phase

A virtual three-dimensional model (VTM) was initially created from the patient’s CT images using dedicated software (Mimics Innovation Suite 21.0; Materialise, Belgium).

CT images for the virtual colonoscopy were acquired after the oral administration of Gastrografin, with an acquisition slice thickness of 1 mm.

**Figure 3:**
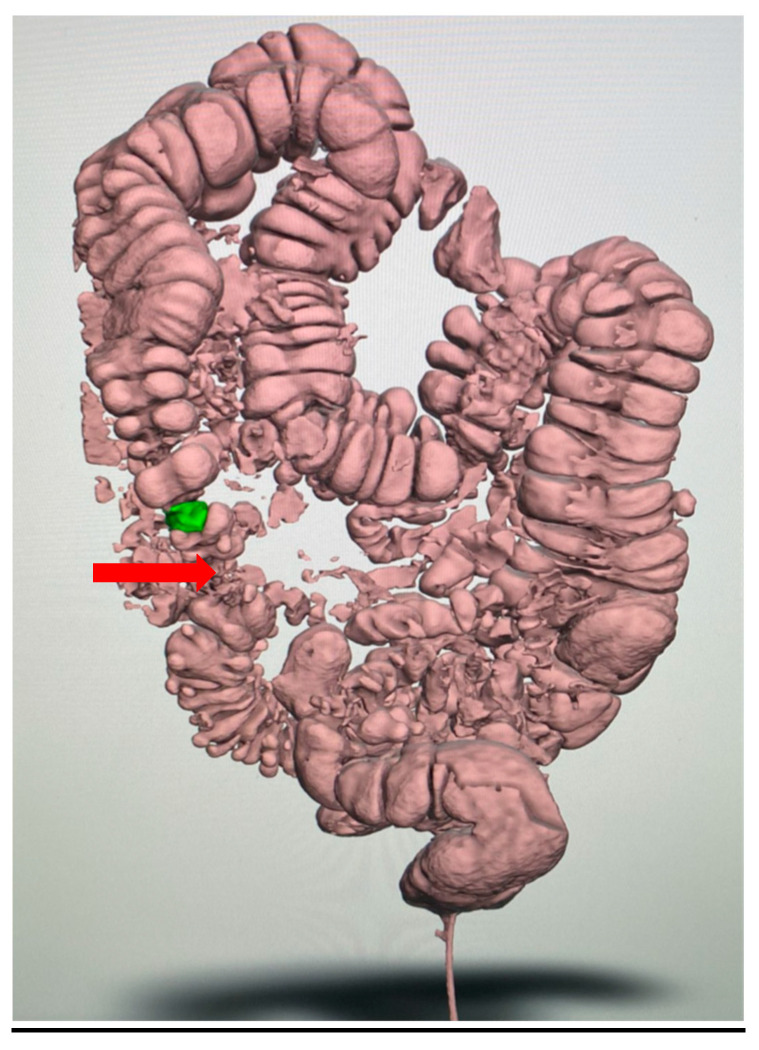
Initial virtual three-dimensional model (VTM) of the colon: The reconstruction illustrates the overall abdominal anatomy and the segmental distribution of diverticular disease (arrow), providing the framework for further segmentation. The stenotic area is highlighted in green.

**Figure 4:**
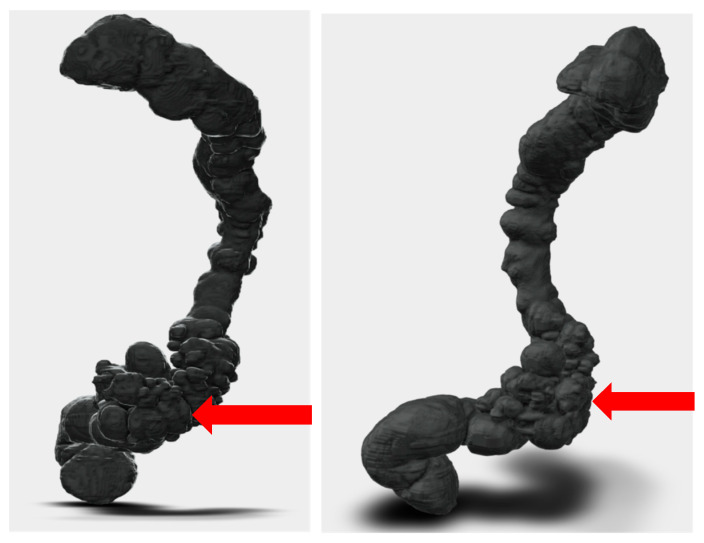
Segmentation and STL files: Segmentation process and preparation of print-ready STL files. The purified digital model highlights the diseased sigmoid colon.

**Figure 5:**
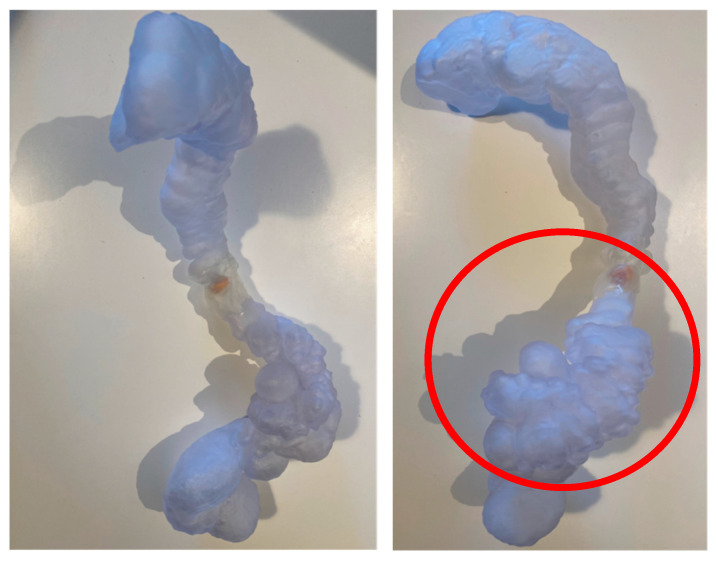
Patient-specific 3D-printed model: Patient-specific 3D-printed model of the colon used for preoperative planning. The pathological segment is highlighted (in the red circle), allowing the surgical team to anticipate the need for left hemicolectomy and possible mobilization of the splenic flexure.

The radiological images, in Digital Imaging and Communications in Medicine (DICOM) format, were imported into the software. The segmentation was then performed to convert the 2D CT images into a single 3D image. Initially, a VTM of the entire abdomen was created (Figure 3) and then exported into multiple Standard Triangulation Language (STL) files, one for each specific anatomical district (Figure 4). The VTM provides surgeons with additional information, offering a more intuitive visualization of the relationships between structures, although the limitations of a purely digital resource are well known. The 3D model was then printed based on these three-dimensional images (Figure 5).

The 3D printing process used was stereolithography (SLA). SLA, or stereolithography, is an additive manufacturing technique that employs a laser beam to selectively polymerize a photosensitive liquid resin layer by layer, solidifying it to create the desired three-dimensional object. Different printing technologies and materials were employed, Visijet (3D Systems, Rock Hill, SC, USA) and Clear and Model resins (Formlabs, Somerville, MA, USA), which ensured an adequate rigidity and transparency for anatomical representation. The design and production of the 3D print were carried out with the assistance of a 3D printing and manufacturing company (3DiFic Srl, Perugia, Italy). The segmentation and design of the model were reviewed for accuracy by both the surgeon and the engineer.

## 3. Discussion

To overcome the limitations of two-dimensional, flat anatomical representations, three-dimensional virtual reconstructions of conventional cross-sectional imaging (MDCT) have been developed with the ultimate goal of facilitating a more detailed understanding [12,13]. Virtual 3D images can be rotated to obtain various viewing angles, enhancing depth perception and improving spatial orientation. However, virtual 3D models are only accessible via a computer and dedicated software. Additionally, assessing relationships and distances between structures can be challenging, as they strongly depend on the model’s orientation on the screen [13]. These limitations can be addressed with 3D printing technology, a tool increasingly applied to surgical planning and training. In gastrointestinal surgery more broadly, patient-specific 3D reconstructions support tailored preoperative planning [14]. This is consistent with recent evidence in colorectal surgery training [15]. In our case, the 3D reconstruction and printed model were directly integrated into preoperative planning. The model allowed the surgical team to anticipate the need for a left hemicolectomy, mobilization of the splenic flexure, and potential ligation of the inferior mesenteric artery. It was also used to rehearse trocar placement and to anticipate potential vascular dissection, thereby providing the surgical team with a clearer understanding of the intraoperative steps. Importantly, the anatomical features predicted by the model—including the extension of diverticulosis and the presence of a stenotic segment—were confirmed intraoperatively, demonstrating a high anatomical fidelity. Although it was not employed as a full-scale simulation or “dry run,” the model offered practical guidance for structuring the mobilization and resection phases and contributed to a greater surgical confidence. Comparable findings have been reported in clinical applications of 3D printing to a laparoscopic colectomy [16].

Although three-dimensional printing was first described more than three decades ago, its use in digestive surgery has expanded significantly in recent years [17]. One of the key benefits of 3D printing is the ability to create patient-specific preoperative models for studying surgical anatomy. Hamabe et al. [18] developed a 3D-printed pelvic model to enhance anatomical understanding during laparoscopic surgery for rectal cancer. Similarly, Garcia-Granero et al. [19] proposed a 3D model for preoperative planning, studying right colon vascularization—specifically the superior mesenteric and ileocolic vessels—to facilitate lymphadenectomy during a complete mesocolic excision for right colon cancer. One major advantage of 3D modeling, based on CT image processing, is the ability to visualize interspatial relationships between anatomical structures in the surgical area of interest. In minimally invasive surgery—both laparoscopic and robotic—the surgeon loses the ability to obtain tactile feedback from manipulated structures. A 3D model can help the surgeon preoperatively recover this tactile perception [13]. The availability of a preoperative 3D model allows for better surgical planning, enabling the evaluation of different approaches, defining dissection planes, and structuring the various phases of the procedure before performing it. This contributes to a reduction in operating time [20]. Advances in 3D printing technology now allow for the creation of models from soft, deformable materials that mimic human tissue properties [8,21,22]. Three-dimensional printing can also be used to simulate real surgeries, as demonstrated by Rundstedt et al. [23]. They described their experience with robot-assisted laparoscopic partial nephrectomy in patients with renal tumors, where preoperative tests were conducted using patient-specific 3D silicone models. Their study highlighted how 3D printing can aid both in surgical decision-making and preoperative simulation, as well as serve as a valuable tool for surgical training.

Combined approaches that integrate 3D reconstruction/printed models with virtual reality further enhance teaching outcomes [24]. Similar findings were reported by Pugliese et al., who described their experience with preoperative studies on 3D models [21]. Based on our experience, we agree with these authors in stating that 3D printing is a significant aid in surgical planning. In our case, the 3D model enabled us to plan a left hemicolectomy instead of a simple sigmoidectomy, ensuring a more appropriate resection for the patient’s diverticular disease. The model also helped anticipate the need to lower the left colic flexure and ligate the inferior mesenteric artery at its origin. Another important application of 3D printing is medical education. The literature suggests that 3D models enhance learning for medical students and surgical residents by providing a tangible, interactive understanding of anatomy. A review by Papazarkadas et al. (2019) [3] also highlighted that patient-specific 3D models can complement operative planning and even serve as perioperative navigation tools. The review further emphasized the promising role of 3D models in surgical training, particularly for complex pathologies [24].

Patient-specific 3D models can also enhance preoperative counseling, improving shared decision-making and alleviating anxiety [25].

Despite its advantages, 3D printing has limitations, primarily related to time and cost. The printing process—from image processing to model production—can take anywhere from a few hours to several days [26,27,28]. Naturally, the more complex the model, the longer it takes to produce. Costs also vary depending on the materials used and the size of the model.

## 4. Conclusions

Based on our review of the literature and our own experience, we can conclude that 3D printing serves as a valuable tool for surgeons in preoperative planning. Its educational benefits are also evident, as it provides realistic anatomical models to enhance understanding. However, due to time and cost constraints, the use of 3D printing in digestive surgery is currently limited to scientific studies rather than routine clinical practice

## Figures and Tables

**Table 1:** Comparison between virtual colonoscopy and 3D-printed model findings.

Feature Evaluated	Virtual Colonoscopy Findings	3D-Printed Model Findings
**Extent of diverticulosis**	Massive diverticulosis of sigmoid and descending colon	Same finding confirmed with clearer visualization of segmental involvement
**Stenotic area**	Suspected narrowing, poorly defined	Stenotic segment clearly delineated, with precise localization and extension
**Anatomical relationships**	Limited perception of spatial relations	Improved understanding of relationship between diseased colon and surrounding structures
**Surgical planning implications**	Suggestive of need for resection	Supported decision for left hemicolectomy
**Tactile/spatial perception**	2D visualization only	Physical 3D model provided tactile and spatial feedback useful for team discussion and planning

## Data Availability

The original contributions presented in this study are included in the article. Further inquiries can be directed to the corresponding author.

## Abbreviations

The following abbreviations are used in this manuscript:

CT	Computed Tomography
DICOM	Digital Imaging and Communications in Medicine
MDCT	Multi-Detector Computed Tomography
STL	Standard Triangulation Language
VTM	Virtual Three-Dimensional Model
